# Virulence, Resistance, and Genomic Fingerprint Traits of *Vibrio cholerae* Isolated from 12 Species of Aquatic Products in Shanghai, China

**DOI:** 10.1089/mdr.2020.0269

**Published:** 2020-12-03

**Authors:** Huiyu Fu, Pan Yu, Weili Liang, Biao Kan, Xu Peng, Lanming Chen

**Affiliations:** ^1^ Key Laboratory of Quality and Safety Risk Assessment for Aquatic Products on Storage and Preservation (Shanghai), China Ministry of Agriculture, College of Food Science and Technology, Shanghai Ocean University, Shanghai, People's Republic of China.; ^2^ National Institute for Communicable Disease Control and Prevention, Chinese Center for Disease Control and Prevention, Beijing, People's Republic of China.; ^3^ Archaea Centre, Department of Biology, University of Copenhagen, Copenhagen, Denmark.

**Keywords:** *Vibrio cholerae*, aquatic products, virulence, antimicrobial susceptibility, heavy metal tolerance, genotyping

## Abstract

*Vibrio cholerae* is a waterborne bacterium and can cause epidemic cholera disease worldwide. Continuous monitoring of *V. cholerae* contamination in aquatic products is imperative for assuring food safety. In this study, we determined virulence, antimicrobial susceptibility, heavy metal tolerance, and genomic fingerprints of 370 *V. cholerae* isolates recovered from 12 species of commonly consumed aquatic products collected from July to September of 2018 in Shanghai, China. Among the species, *Leiocassis longirostris*, *Ictalurus punetaus*, *Ophiocephalus argus Cantor*, and *Pelteobagrus fulvidraco* were for the first time detected for *V. cholerae*. Toxin genes *ctxAB*, *tcpA*, *ace*, and *zot* were absent from all the *V. cholerae* isolates. However, high occurrence of virulence-associated genes was detected, such as *hapA* (82.7%), *hlyA* (81.4%), *rtxCABD* (81.4%, 24.3%, 80.3%, and 80.8%, respectively), and *tlh* (80.5%). Approximately 62.2% of the 370 *V. cholerae* isolates exhibited resistance to streptomycin, followed by ampicillin (60.3%), rifampicin (53.8%), trimethoprim (38.4%), and sulfamethoxazole-trimethoprim (37.0%). Moreover, ∼57.6% of the isolates showed multidrug resistant phenotypes with 57 resistance profiles, which was significantly different among the 12 species (multiple antimicrobial resistance index, *p* < 0.001). Meanwhile, high incidence of tolerance to heavy metals Hg^2+^ (69.5%), Ni^2+^ (32.4%), and Cd^2+^ (30.8%) was observed among the isolates. The enterobacterial repetitive intergenic consensus-polymerase chain reaction (ERIC-PCR)-based fingerprinting profiles classified the 370 *V. cholerae* isolates into 239 different ERIC-genotypes, which demonstrated diverse genomic variation among the isolates. Overall, the results in this study meet the increasing need of food safety risk assessment of aquatic products.

## Introduction

V*ibrio* cholerae is a Gram-negative bacterium that is autochthonously inhabited in aquatic environments worldwide. The bacterium can cause cholera, a severe diarrheal disease that is typically transmitted via contaminated water and person-to-person contact.^1,2^ It is estimated that *V. cholera* caused roughly 2.9 million cases of cholera and 95,000 deaths annually worldwide between 2008 and 2012.^3^ In recent years, cholera remained endemic in developing countries, and the most recent outbreak was reported in Mozambique in March 27, 2019 (World Health Organization, WHO^†^).

Aquatic animals such as fish, shellfish, and crustaceans are important reservoirs and vectors of *V. cholerae*.^4^ For instance, *V. cholerae* was isolated from ∼30 fish species belonging to 9 different orders within the *Actinopterygii* class.^4^ Recently, pathogenic *V. cholerae* (serotype: O1) was found in *Rastrineobola argentea* in Lake Victoria, Tanzania in 2017.^5^ Thus, continuous monitoring of aquatic products and identification of risk factors of *V. cholerae* are imperative for assuring food safety.

Epidemic *V. cholerae* strains (serotypes O1 and O139) produce cholera toxin (CT) and toxin coregulated pilus (TCP).^6^ Non-O1/O139 *V. cholerae* isolates carrying virulence-associated genes are widely distributed in aquatic ecosystems, and they can cause mild gastroenteritis, cholera-like diarrhea, sepsis, or other extraintestinal infections.^7–12^ For instance, it has been reported that ∼77% of infection cases were caused by nontoxigenic *V. cholerae* in Northern Europe.^13^ Previous studies have revealed virulence-associated factors involved in the pathogenicity of *V. cholerae*, including zonula occludens toxin (*zot*), accessory cholera enterotoxin (*ace*), El Tor hemolysin (*hlyA*), hemagglutinin protease (*hapA*), RTX toxin (*rtxCABD*), thermolabile hemolysin (*tlh*), mannose-sensitive hemagglutination (MSHA; *mshA*), and putative type IV pilus (*pilA*).^14–18^ Therefore, continuous detection of the non-O1/0139 *V. cholerae* in aquatic products is also crucial for food safety systems.

Antimicrobial agents are widely applied in aquaculture industry for effective control of pathogenic bacteria; nevertheless, the indiscriminate use of antibiotics leads to the emergence of antibiotic-resistant bacteria, particularly in developing countries.^19^ Resistant *V. cholerae* isolates that originated from clinical and environmental sources have been reported.^18,20–24^ For example, during an outbreak of cholera in Guinea-Bissau in 1996–1997, case-fatality rates increased from 1% to 5.3% after the emergence of multidrug-resistant (MDR) *V. cholerae*.^20^ Chomvarin *et al.* reported that *V*. *cholerae* O1 strains (*n* = 35) isolated from clinical samples and natural surface water sources in Thailand were resistant to trimethoprim (TM)/sulfamethoxazole and/or tetracycline (TET)/or ampicillin (AMP).^21^

Recently, Verma *et al.* reported that 99% of *V. cholerae* strains (*n* = 438) from stool samples of diarrheal patients in India were resistant to more than two antibiotics.^24^ Lepuschitz *et al.* investigated *V. cholerae* isolates (*n* = 82) recovered from an Austrian lake, and they found that the majority of the isolates showed resistance to sulfonamides (*n* = 80), and some to streptomycin (STR; *n* = 32) and AMP (*n* = 17).^23^ Xu *et al.* reported that ∼65.3% of *V. cholerae* isolates (*n* = 400) from four species of commonly consumed fish in Shanghai, China were resistant to STR, followed by AMP (44.5%) and rifampicin (RIF; 24.0%).^18^

Heavy metals used in mining, fossil fuel combustion, and agricultural practices can enter the food chain, such as cadmium (Cd), chromium (Cr), mercury (Hg), and Pb.^25^ For instance, Li and Xie examined heavy metal contents in eleven species of fish collected from the Three Gorges Reservoir in China, and they found that the concentrations of Hg in carnivorous fish were higher than those in omnivorous fish.^26^ Vu *et al.* analyzed heavy metal concentrations of five species of fish in the Houjing River in Taiwan, and they found that heavy metal concentrations of arsenic (As), Cd, Cr, copper (Cu), nickel (Ni), and Pb were higher than officially permissible limits.^27^ Recently, Xu *et al.* also reported that ∼49.3% of the *V. cholerae* isolates (*n* = 400) were tolerant to Hg^2+^, followed by Zn^2+^ (30.3%), and Pb^2+^ (12.0%).^18^

The People's Republic of China is the world's largest producer, consumer, and exporter of aquatic products. For instance, *Scophthalmus maximus* (known as turbot) is one of the most important economic marine flatfish species in Europe, which was introduced to China in the 1990s, and its production reached 640,000 tons in 2013 in China.^28^ Among crustaceans, the largest output was yielded from *Penaeus vannamei* with 1,672,287 tons in 2017 in China.^29^ In addition, *Ostrea gigas thunberg* is the world's widely cultured shellfish, and its production reached 4,879,422 tons in 2017 in China.^29^

In our prior studies, we surveyed prevalence traits of *V. cholerae* strains isolated from four species of fish, three species of shrimps, and water samples from the Yangtze River Estuary in 2011–2017.^18,30,31^ In this study, we determined virulence, resistance, and genetic diversity of 370 *V. cholerae* isolates recovered from 12 species of aquatic products, including nine species of fish, one species of crustacean, and two species of shellfish (see the Materials and Methods section). To our knowledge, four species of fish thereof were for the first time monitored for *V. cholerae*, including *Ictalurus punetaus*, *Leiocassis longirostris*, *Ophiocephalus argus Cantor*, and *Pelteobagrus fulvidraco*. This study meets the increasing need for decreasing and controlling pathogenic *V. cholerae* persistence in aquatic products.

## Materials and Methods

### Sample collection

The 12 species of commonly consumed aquatic products included 9 species of fish: *Aristichthys nobilis*, *Carassius auratus*, *Ctenopharyngodon idellus*, *I. punetaus*, *L. longirostris*, *O. argus Cantor*, *P. fulvidraco*, *Parabramis pekinensis*, *S. maximus*; 1 species of crustacean: *P. vannamei*; and 2 species of shellfish: *O. gigas thunberg* and *Placopecten magellanicus*. The samples were collected in two large aquatic product markets located in Shanghai, China, including the Jiangyang Aquatic Market (31°21′25.90′′N, 121°26′50.68′′E) and Oriental International Aquatic Market (31°20′6.76′′N, 121°32′17.68′′E), during July to September in 2018. A total of 92 samples, including fish (*n* = 50), crustacean (*n* = 30), and shellfish (*n* = 12), were collected in sterile sampling bags (Nanjing Maojie Microbial Technology Co., Ltd., Nanjing, China), and they were immediately transported in an ice box (700 × 440 × 390 mm) to the laboratory at Shanghai Ocean University in Shanghai for analysis. This study did not include epidemic *V. cholerae* strains.

### Identification of *V. cholerae* isolates

*V. cholerae* was identified by biochemistry and molecular biology methods as described in our previous study.^18^ Briefly, properly diluted samples were spread on to selective thiosulfate citrate bile salts sucrose (TCBS, pH 8.5, 3.0% NaCl; Beijing Land Bridge Technology Co., Ltd., Beijing, China) agar plates, and they were incubated at 37°C for 16–18 hr. Yellow colonies were considered to be presumptive *V. cholerae*, and they were then inoculated into Double-Arginine Hydrolase Test Medium and Esculin Medium (Muwei Biotechnology Co., Ltd., Shanghai, China) for the arginine dihydrolase test and esculin hydrolysis test, respectively. *V. cholerae* isolates showing negative reactions in both two biochemical tests were further identified by polymerase chain reaction (PCR) assays. *V. cholerae* GIM 1.449 (Guangdong Culture Collection Center, Guangzhou, China) was used as a positive control strain.

*V. cholerae*-specific *lolB* and bacterial 16S ribosomal RNA (rRNA) genes were detected by PCR assays as previously described.^18,32^ Genomic DNA was extracted by using a thermal lysis method.^18^ A 20 μL of PCR reaction mixture contained 8 μL of DNase/RNase-free deionized water (Tiangen Biotech Co., Ltd., Beijing, China), 10 μL of 2 × Taq Master Mix (Novoprotein Technology Co., Ltd., Shanghai, China), 0.5 μL of each primer (5 μM) (Table 1), and 1 μL of genomic DNA. PCR reactions were performed by using Mastercycler^®^ pro PCR thermal cycler (Eppendorf, Hamburg, Germany) under the conditions as previously described.^18^ Amplicons were analyzed by agarose gel electrophoresis, visualized, and recorded by using a UVP EC3 Imaging system (UVP, LLC, Upland, CA) as previously described.^18^

**Table 1.** Oligonucleotide Primers Used in This Study

Primer	Sequence (5′-3′)	PAS (bp)	AT (*°*C)	References
27F	GAGAGTTTGATCCTGGCTCAG	1,500	55	^63^
1492R	TACGGCTACCTTGTTACGAC			
*ace*-F	GCTTATGATGGACACCCTTTA	284	55	^64^
*ace*-R	GTTTAACGCTCGCAGGGCAAA			
*ctxAB*-F	TGAAATAAAGCAGTCAGGTG	778	55	^65^
*ctxAB*-R	GGTATTCTGCACACAAATCAG			
ERIC1R	ATGTAAGCTCCTGGGGATTCAC		52	^61^
ERIC2	AAGTAAGTGACTGGGGTGAGCG			
*hapA*-F	CGTTAGTGCCCATGAGGTC	207	55	^18^
*hapA*-R	CGTGACGGCTGATCGAAAT			
*hlyA*-F	CAATCGTTGCGCAATCGCG	265	50	^34^
*hlyA*-R	TTGACCTTCAGCATCACT			
*mshA*-F	CGCTAGATACTTCGAGTGAG	189	52	^18^
*mshA*-R	TACCACAAGCAGTTCCAG			
*pilA*-F	GCGATTGCAATTCCTCAA	227	53	^18^
*pilA*-R	CCTAATGCACCTGATGCT			
*rtxA*-VC1451F	GATTCTTCCGTTCAAGCTCCG	2,571	58	^11^
*rtxA*-1451R	TGGTTCAGGCTGTTGCACAC			
*rtxB*-F	ATTCATTTTTATTTAAGTGTCATCA	400	50	^18^
*rtxB*-R	TTTCGCTCAGCACTCTTT			
*rtxC*-F	ATGTCTATTACACATCAACCTGCAA	437	54	^18^
*rtxC*-R	CGGATACAGCGGTCATTT			
*rtxD*-F	ATCATGAAGCGTTTCTTTGGTCAAA	334	58	^18^
*rtxD*-R	CGCCCAAGGTATCAAGAGTCAG			
*tcpA*-F	ATGCAATTATTAAAACAGCTTTTTAAG	675	54	^33^
*tcpA*-R	TTAGCTGTTACCAAATGCAACAG			
*tlh*-F	TGGGAGTGGGCAAAGAAT	274	53	^18^
*tlh*-R	AAAGGCTATCGCCAAACG			
VHMF	TGGGAGCAGCGTCCATTGTG		57	^32^
VHA-AS5	CAATCACACCAAGTCACTC	516		
*zot*-225F	TCGCTTAACGATGGCGCGTTTT	947	55	^66^
*zot*-1129R	AACCCCGTTTCACTTCTACCCA			

AT, annealing temperature; PAS, predicted amplicon size.

The PCR products were validated by DNA sequencing at Shanghai Sangon Biological Engineering Technology and Services Co., Ltd. (Shanghai, China). Sequence analysis was performed by using Basic Local Alignment Search Tool (BLAST) software against the GenBank database.

### Detection of virulence and virulence-associated genes

The major virulence genes (*ctxAB* and *tcpA*) (Kumar *et al.* )^33^ and virulence-associated genes (*ace*, *zot*, *rtxABCD*, *hapA*, *hlyA*, *tlh*, *mshA*, and *pilA*).^6,11,18,34^ were detected by PCR assay using the primer pairs listed in Table 1. The PCR reaction conditions varied depending on the melting temperature (Tm) values of the primer pairs. All the oligonucleotide primers used in this study were synthesized by Sangon. The genomic DNA of *V. cholerae* ATCC39315 (N16961) was used as a positive control.

### Antibiotic susceptibility and heavy metal tolerance assays

*V. cholerae* isolates were measured for *in vitro* susceptibility to 10 antimicrobial agents, and tolerance to 8 heavy metals according to the methods described in our previous studies.^18,35^ The antimicrobial agents (Oxoid) included 10 μg AMP, 30 μg chloramphenicol (CHL), 10 μg STR, 10 μg gentamicin (CN), 30 μg kanamycin (KAN), 5 μg RIF, 100 μg spectinomycin (SPT), 30 μg TET, 5 μg TM, and 25 μg SXT (sulfamethoxazole [23.75 μg]-trimethoprim [1.25 μg]). The heavy metals included CdCl_2_, CrCl_3_, CuCl_2_, PbCl_2_, HgCl_2,_ NiCl_2_, MnCl_2_, and ZnCl_2_ (analytical reagents; Sinopharm Chemical Reagent Co., Ltd., Shanghai, China) with concentrations tested in the range from 3.125 to 3,200 μg/mL. *Escherichia coli* strains ATCC25922 and K12 (Institute of Industrial Microbiology, Shanghai, China) were used as quality control strains.^18,35^

### Enterobacterial repetitive intergenic consensus-PCR assay

The enterobacterial repetitive intergenic consensus-PCR (ERIC-PCR) was performed according to a method previously described.^18^ The ERIC-PCR reactions were performed under the following conditions: initial denaturation at 95°C for 8 min, followed by 32 cycles of 94°C for 30 sec, 52°C for 1 min, and 65°C for 8 min, and a final extension at 65°C for 16 min. The ERIC-PCR products (6 μL/sample) were analyzed by electrophoresis at 120 V for about 30 min on a 1.0% agarose gel. Amplified DNA fragments were visualized and recorded as described earlier. DNA banding patterns generated by the ERIC-PCR were clustered by the unweighted pair group method with arithmetic mean (UPGMA) by using the Dice coefficient.^18^ A dendrogram was constructed by using BioNumeric software v.7.6.^6^ Simpson's index of diversity was calculated as previously described.^36^

### Statistical analysis

Data analysis was performed by using SPSS software version 17.0 (SPSS, Inc., Chicago, IL). The multiple antimicrobial resistance index (MARI) of isolates was defined as previously described.^37^ The MARI is often used to determine the antibiotic resistance-associated health risk. Statistically significant differences between aquatic products and MARI of resistant isolates were determined by one-way analysis of variance followed by appropriate *post hoc* text (Turkey).^18^

## Results

### Prevalence of *V. cholerae* isolates in 12 species of aquatic products

In this study, *V. cholerae* strains were isolated from 92 samples of aquatic products, including 9 species of fish, 1 species of crustacean, and 2 species of shellfish. Approximately 3,376 yellow single colonies were randomly selected from the selective TCBS agar plates for further identification. Approximately 30.5% (1,028/3,376) of the yellow colonies were detected negative for the arginine dihydrolase activity test and esculin hydrolysis test, and they were positive for the *V. cholerae*-specific gene *lolB*. The results were confirmed by DNA sequencing of the *lolB* and 16S rRNA genes. Approximately 95.6% (983/1,028) of the *V. cholerae* isolates were recovered from the fish, 3.2% (33/1,028) from the crustacean, and 1.2% (12/1,028) from the shellfish samples. A pure culture of randomly selected 370 *V. cholerae* isolates was further analyzed and reported in this study, including 328 isolates from the fish (*A. nobilis*, *C. auratus*, *C. idellus*, *O. argus Cantor*, *I. punetaus*, *L. longirostris*, *P. fulvidraco*, *P. pekinensis* and *S. maximus*), 12 isolates from the shrimp (*P. vannamei*), and 30 isolates from the shellfish (*O. gigas thunberg* and *P. magellanicus*) samples.

### Virulence and virulence-associated gene profiles in the *V. cholerae* isolates

**FIG. 1.**
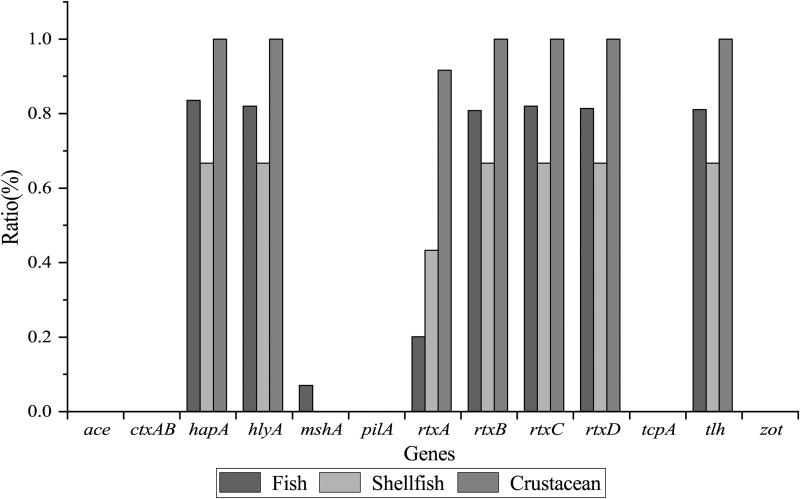
Virulence and virulence-associated genes of the 370 *Vibrio cholerae* isolates.

Virulence and virulence-associated gene profiles of the 370 *V. cholerae* isolates were obtained (Fig. 1). All the *V. cholerae* isolates were detected negative for the toxin genes *ctxAB* and *tcpA*. Moreover, none of the isolates carried the virulence-associated genes *ace*, *zot*, and *pilA*. In contrast, the *hapA* (82.7%), *hlyA* (81.4%), *rtxBCD* (80.3%, 81.4%, and 80.8%, respectively), and *tlh* (80.5%) genes were prevalent in the 370 *V. cholerae* isolates, ∼24.3% of which also carried the *rtxA* gene. A low percentage of the *mshA* gene (6.0%) was observed among the isolates.

As shown in Fig. 1, the *V. cholerae* isolates in the three types of aquatic products had different virulence genotypes. The *mshA* gene was only present in *V. cholerae* isolates that originated from the fish samples; whereas the *hapA*, *hlyA*, *rtxCABD*, and *tlh* genes were detected positive in the isolates from the fish, crustacean, and shellfish with different percentages. Among the *V. cholerae* isolates in fish, the *hapA* gene accounted for the highest proportion (83.5%), followed by *rtxC* (82.0%) and *hlyA* (82.0%). The occurrence of the *hapA*, *hlyA*, *rtxBCD*, and *tlh* genes was observed to be similar in the shrimp isolates (66.7%). All the *V. cholerae* isolates in shellfish were detected positive for the *hapA* (100%), *hlyA* (100%), *rtxBCD* (100%), and *tlh* (100%) genes, most of which also carried the *rtxA* gene (91.7%). In contrast, a lower incidence of the *rtxA* gene was observed in the fish (19.9%) and shrimp (43.3%) isolates.

As shown in Table 2, all the 370 *V. cholerae* isolates were classified into 17 different virulence gene profiles. Among these, the *hapA^+^/hlyA^+^/tlh^+^/rtxB^+^/rtxC^+^/rtxD^+^* profile was the most predominant (55.4%) in the isolates, followed by the *hapA^+^/hlyA^+^/tlh^+^/rtxA^+^/rtxB^+^/rtxC^+^/rtxD^+^* (16.2%), and *hapA^+^/hlyA^+^/mshA^+^/tlh^+^/rtxA^+^/rtxB^+^/rtxC^+^/rtxD^+^* (5.4%). In addition, about 1.4% of the isolates had unique profiles (*hapA^+^*, *hlyA^+^*). In contrast, ∼16.5% (*n* = 61) of the isolates harbored no virulence-associated genes tested in this study.

**Table 2.** Virulence and Virulence-Associated Gene Profiles of the 370 *Vibrio cholerae* Isolates

No. of genes	Genotype	No. of isolates
0	—	61
1	*hapA^+^*	3
	*hlyA^+^*	2
2	*hapA^+^/hlyA^+^*	2
3	*hapA^+^/hlyA^+^/rtxC^+^*	1
	*hapA^+^/hlyA^+^/rtxD^+^*	2
4	*hapA^+^/rtxB^+^/rtxC^+^/rtxD^+^*	2
	*hlyA^+^/rtxB^+^/rtxC^+^/rtxD^+^*	1
	*hapA^+^/hlyA^+^/rtxC^+^/rtxD^+^*	1
	*hapA^+^/hlyA^+^/tlh^+^/rtxC^+^*	1
5	*hapA^+^/hlyA^+^/rtxB^+^/rtxC^+^/rtxD^+^*	4
	*hapA^+^/tlh^+^/rtxB^+^/rtxC^+^/rtxD^+^*	1
6	*hapA^+^/hlyA^+^/tlh^+^/rtxA^+^/rtxB^+^/rtxC^+^*	1
	*hapA^+^/hlyA^+^/tlh^+^/rtxB^+^/rtxC^+^/rtxD^+^*	205
7	*hapA^+^/hlyA^+^/tlh^+^/rtxA^+^/rtxB^+^/rtxC^+^/rtxD^+^*	60
	*hapA^+^/hlyA^+^/mshA^+^/tlh^+^/rtxB^+^/rtxC^+^/rtxD^+^*	3
8	*hapA^+^/hlyA^+^/mshA^+^/tlh^+^/rtxA^+^/rtxB^+^/rtxC^+^/rtxD^+^*	20

### Antimicrobial resistance profiles of the *V. cholerae* isolates

**FIG. 2.**
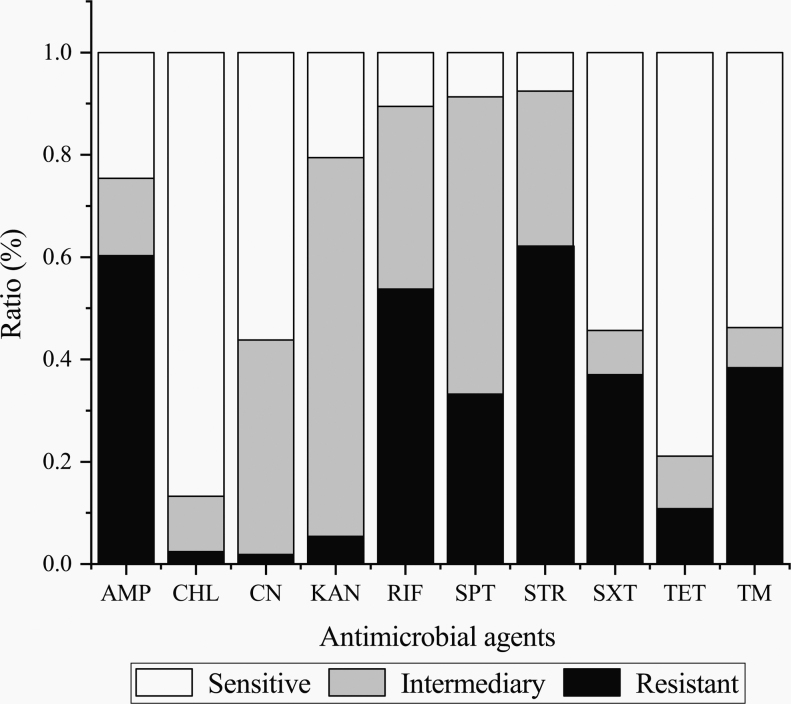
Antimicrobial susceptibility of the 370 *Vibrio cholerae* isolates. AMP, ampicillin; CHL, chloramphenicol; CN, gentamicin; KAN, kanamycin; RIF, rifampicin; SPT, spectinomycin; STR, streptomycin; SXT, sulfamethoxazole-trimethoprim; TET, tetracycline; TM, trimethoprim.

Antimicrobial susceptibility of the 370 *V. cholerae* isolates to 10 antimicrobial agents was determined (Fig. 2). The results showed that more than half of the isolates were resistant to STR (62.2%, *n* = 230), AMP (60.3%, *n* = 223), and RIF (53.8%, *n* = 199). Moreover, ∼74.1% (*n* = 274) and 58.1% (*n* = 215) of the isolates showed intermediate resistance to KAN and SPT. Notably, ∼24.1% (*n* = 89) of the isolates were resistant to at least 5 antimicrobial agents, among which 2 isolates (*V. c*-*C. idellus* 0802-05, *V. c*-*C. idellus* 0802-08) were resistant to 8 of 10 antimicrobial drugs evaluated in this study. In contrast, the majority of the isolates were susceptible to CHL (86.8%, *n* = 321) and TET (78.9%, *n* = 292), and almost half to CN (56.2%, *n* = 208). Moreover, ∼7.3% (*n* = 27) of the isolates were sensitive to all the 10 antimicrobials agents tested, 5.4% (*n* = 20) of which were recovered from fish samples, 1.6% (*n* = 6) from crustacean, and 0.3% (*n* = 1) from shellfish. The resistance trend of the isolates was STR>AMP>RIF>TM>SXT>SPT>TET>KAN>CHL>CN.

**FIG. 3.**
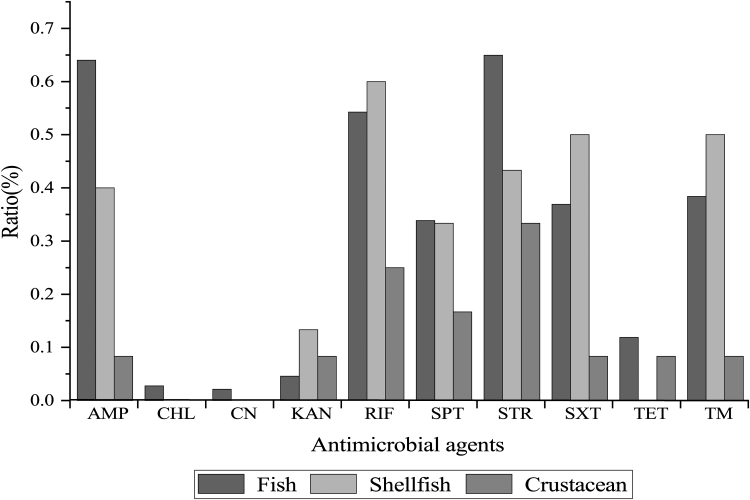
Antimicrobial resistance of the 370 *Vibrio cholerae* isolates in the three types of aquatic products.

As shown in Fig. 3, the *V. cholerae* isolates in the fish, crustacean, and shellfish samples exhibited different antimicrobial susceptibility profiles. The STR resistance was the most prevalent among the 3 types of aquatic products (33.3–64.9%), followed by AMP (8.0–64.0%), RIF (25.0–54.3%), and TM (8.0–38.4%). Meanwhile, the isolates that originated from shellfish had higher resistance incidence to RIF (60.0%), SXT (50.0%), and TM (50.0%) than those from the fish (54.3%, 36.9%, 38.4%) and shrimp (25.0%, 8.3%, 8.3%) samples, respectively. The resistance to CHL (2.7%) and CN (2.1%) was only detected in the isolates from fish. Approximately 11.9% and 8.3% of the isolates from the fish and crustacean were resistant to TET, respectively, whereas none of the isolates from the shellfish was resistant to this drug.

Resistance to STR was the most prevalent (25.0–100%) among the *V. cholerae* isolates that originated from the 12 species of aquatic products, followed by to RIF (12.5–100%). Moreover, approximately 7.4–100% of the isolates from all the species showed resistance to AMP, except the *P. magellanicus*. In contrast, the isolates from all the species were sensitive to CHL, except the *C. auratus.* Also, all the isolates from *P. pekinensis* were sensitive to CHL, TET, SXT, and TM. However, the occurrence of resistant isolates to the later 2 drugs was higher in the other 11 species of aquatic products (SXT: 8.3–85.7%; TM: 8.3–92.9%) (Supplementary Table S1).

In this study, ∼20.3% (*n* = 75) of the *V. cholerae* isolates recovered from the fish samples had MARI values higher than 0.4, showing resistance to five antimicrobial agents tested. Among these isolates, two from *C*. *idellus* (*V. c*-*C. idellus* 0802-05, and *V. c*-*C. idellus* 0802-08) had the maximum MARI of 0.8. In contrast, a lower percentage (3.5%, *n* = 13) of the isolates from shellfish had MARI values >0.4. The results indicated that an obviously higher occurrence of MDR isolates was present in the fish than that in the shellfish and crustacean samples.

### Heavy metal tolerance profiles of the *V. cholerae* isolates

Tolerance of the 370 *V. cholerae* isolates to 8 heavy metals was determined, and the results are presented in Table 3. When compared with the quality control strain *E. coli* K12, the isolates exhibited maximum minimal inhibitory concentration (MIC) values of 1,600 μg/mL for Pb^2+^ and Mn^2+^; 800 μg/mL for Ni^2+^, Cr^3+^, and Cu^2+^; 400 μg/mL for Cd^2+^, Zn^2+^; and 50 μg/mL for Hg^2+^. Approximately 69.5% of the isolates were tolerant to Hg^2+^, followed by Ni^2+^ (32.4%), and Cd^2+^ (30.8%); whereas only a few isolates were resistant to Cu^2+^ (1.1%), Pb^2+^ (0.5%), and Mn^2+^ (0.3%). In contrast, all the isolates were sensitive to Cr^3+^. The tolerance trend of the 370 *V. cholerae* isolates was Hg^2+^>Ni^2+^>Cd^2+^>Zn^2+^>Cu^2+^>Pb^2+^> Mn^2+^>Cr^3+^.

**Table 3.** Tolerance of the 370 *Vibrio cholerae* Isolates to 8 Heavy Metals

Heavy metal	MIC (μg/mL)											Resistant	
	3.125	6.25	12.5	25	50	100	200	400	800	1,600	3,200	n	(%)
Ni^2+^							^a^						
					3	24	223	82	38			120	32.4
Cd^2+^						^a^							
				1	6	249	109	5				114	30.8
Cr^3+^									^a^				
						1	7	119	243			0	0
Cu^2+^								^a^					
					1	34	136	195	4			4	1.1
Pb^2+^									^a^				
							5	8	355	2		2	0.5
Hg^2+^	^a^												
	113	124	91	40	2							257	69.5
Zn^2+^							^a^						
					1	102	241	26				26	7.0
Mn^2+^									^a^				
				3	12	47	222	39	46	1		1	0.3

^a^ MIC of the quality control strain *Escherichia coli* K12.

MIC, minimal inhibitory concentration.

**FIG. 4.**
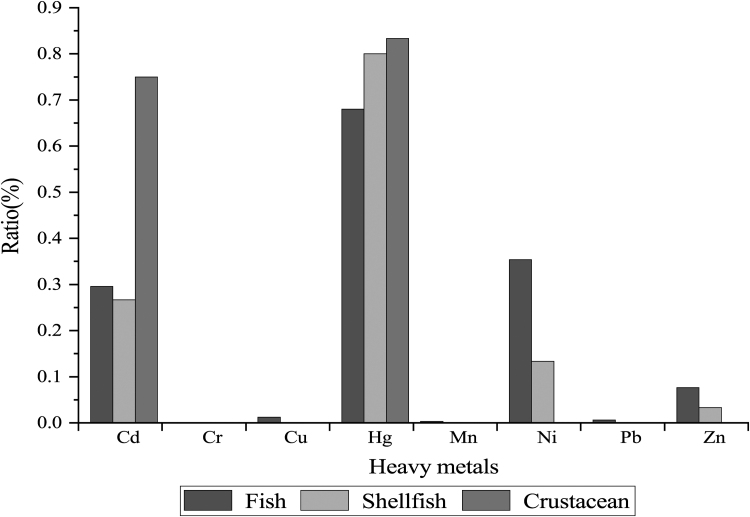
Heavy metal tolerance of the 370 *Vibrio cholerae* isolates in the three types of aquatic products.

The *V. cholerae* isolates in the three types of aquatic products had different heavy metal tolerance profiles (Fig. 4). The highest percentage of tolerance to Hg^2+^ was observed in the crustacean (83.3%), shellfish (80.0%), and fish (68.0%) isolates, followed by Cd^2+^ (shrimp, 75.0%; fish, 29.6%; and shellfish, 26.7%). Moreover, some isolates from the fish (35.4%, 13.3%) and shellfish (7.6%, 3.3%) had tolerance to Ni^2+^ and Zn^2+^, respectively, but none of the shrimp isolates was tolerant to these two heavy metals. Tolerance to Cu^2+^ (1.2%), Mn^2+^ (0.3%), and Pb^2+^ (0.6%) was only observed in the isolates from the fish samples.

Different heavy metal tolerance profiles were also observed among the 12 species of aquatic products (Supplementary Table S2). The isolates from *C. auratus*, *I. punetaus*, *O. argus Cantor*, and *S. maximus* were tolerant to the maximum number of heavy metals (five of eight heavy metals), followed by *C. idellus* and *L. longirostris* (four of eight heavy metals), as well as *A. nobilis*, *P. pekinensis*, *O. gigas thunberg* and *P. magellanicus* (three of eight heavy metals). Moreover, the highest percentage of tolerance to Ni^2+^ was observed in the isolates from *C. idellus* (90.0%) and *L. longirostris* (90.0%), when compared with the isolates from the other species (0–36.3%). In addition, the unique resistance to Mn^2+^ was detected in the isolates from *C. idellus* (3.3%), and to Pb^2+^ it was observed from *C. auratus* (3.3%) and *S. maximus* (1.6%).

### Genomic fingerprints of the *V. cholerae* isolates

Genetic diversity of the 370 *V. cholerae* isolates recovered from the 12 species of aquatic products was evaluated by the ERIC-PCR assay. The obtained fingerprinting profiles comprised various sizes of DNA bands, consistent with a previous report.^18^ On the basis of the fingerprinting profiles, all the isolates were classified into 239 different ERIC-genotypes, and 167 isolates (69.9%) thereof were assigned as singletons. Approximately 94.0% (*n* = 157) and 6.0% (*n* = 10) of these singletons were recovered from the fish and shellfish, respectively, whereas none was recovered from the crustacean samples. The UPGMA algorithm grouped all the 239 ERIC genotypes into 18 distinct clusters at a 73.0% similarity cutoff level (Figure not shown). Most isolates had a similarity coefficient of 50.0–100%, and the Simpson's diversity index was 0.8150. These results demonstrated high genetic diversity of the 370 *V. cholerae* isolates recovered from the 12 species of aquatic products.

In addition, ∼54.9% (*n* = 203) of the 370 *V. cholerae* isolates shared 71 ERIC-genotypes. Among these isolates, ∼84.2% (*n* = 171) were recovered from fish, 9.9% (*n* = 20) from shellfish, and 5.9% (*n* = 12) from crustacean, respectively. For example, nine isolates that shared an ERIC genotype *vc*00012 were derived from *O. argus Cantor* (*n* = 6), *C. auratus* (*n* = 2), and *I. punetaus* (*n* = 1), respectively, suggesting perhaps near phylogenetic relationships among the *V. cholerae* isolates.

### Comparison of the MDR and heavy metal tolerance

**FIG. 5.**
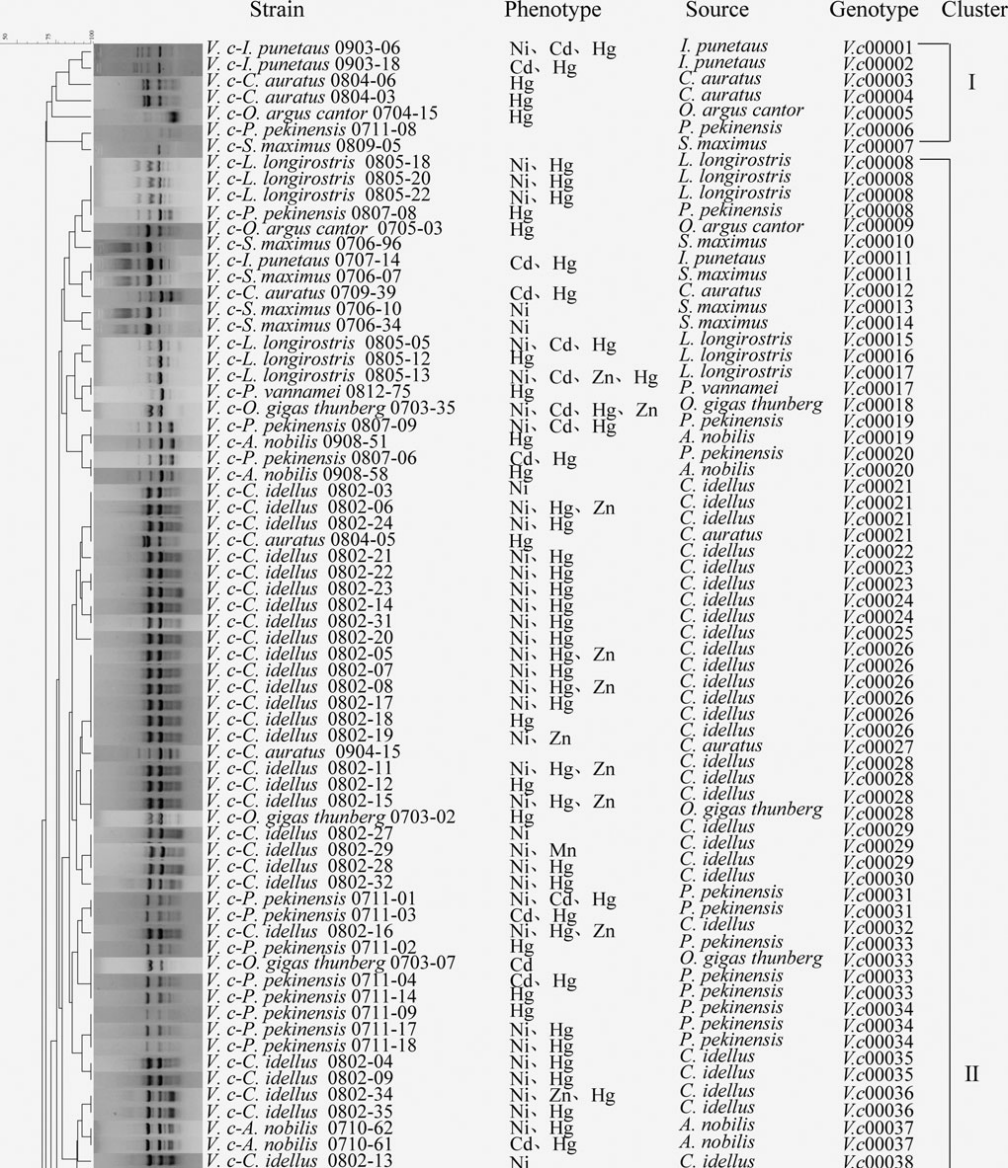

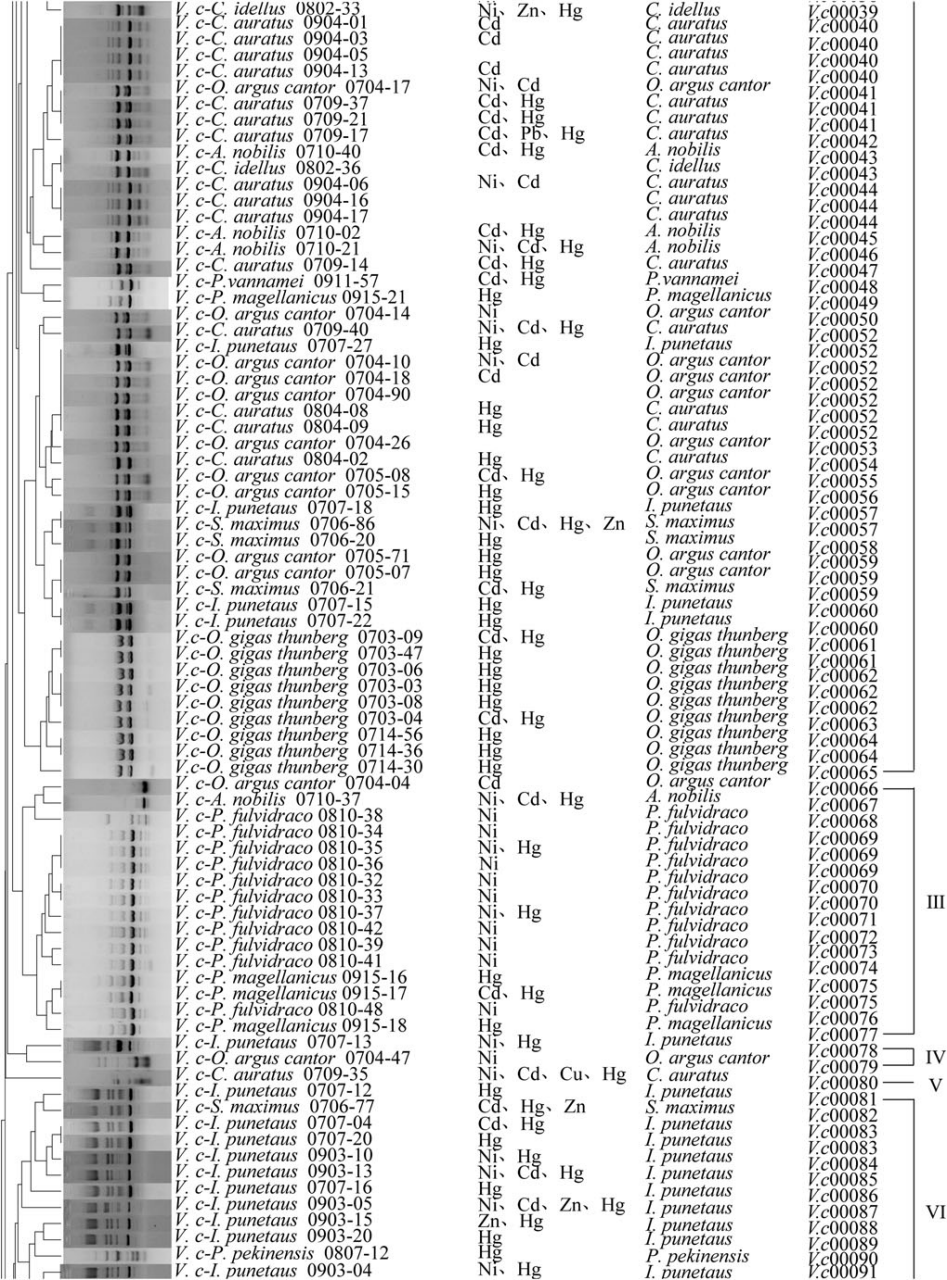

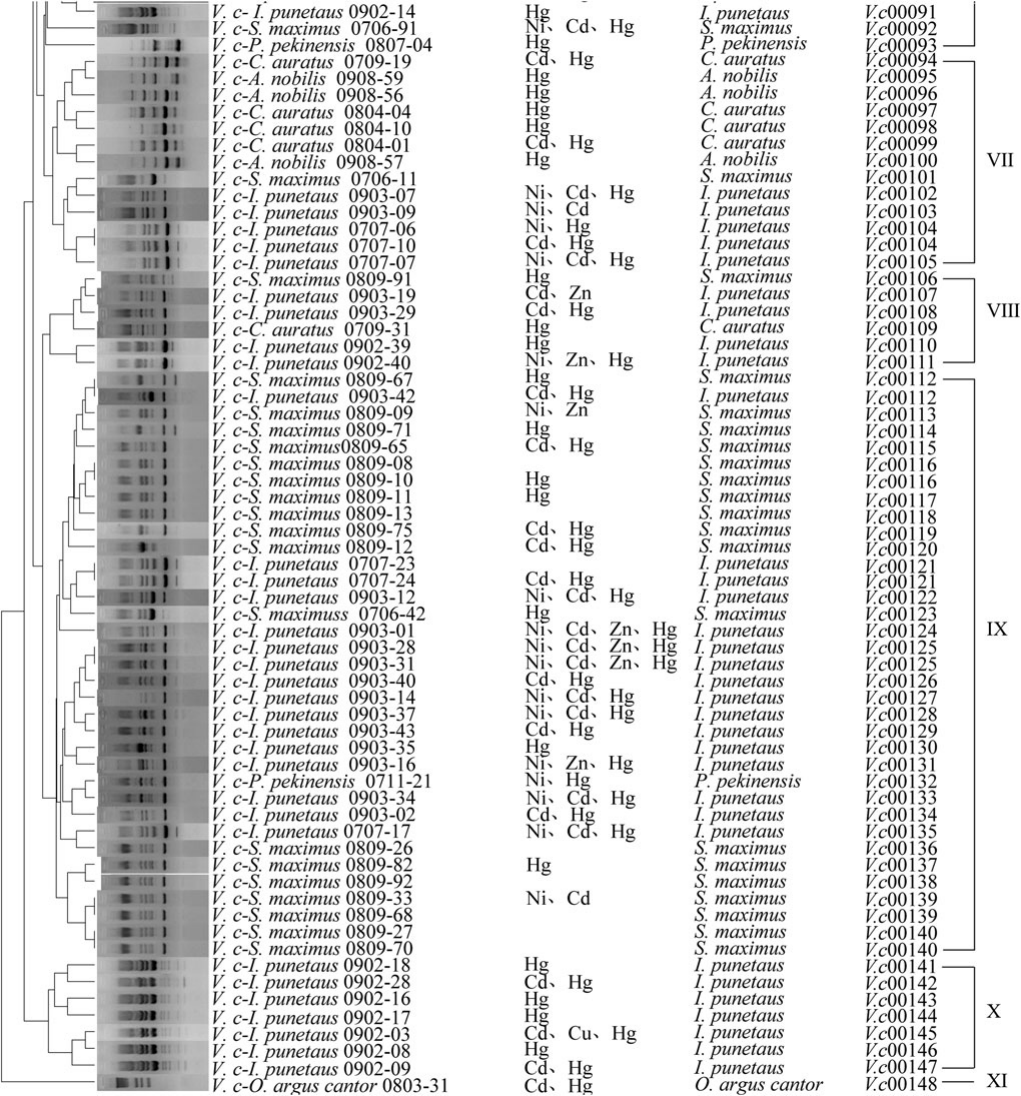
The ERIC-PCR fingerprinting profiles of the MDR *Vibrio cholerae* isolates. ERIC-PCR, enterobacterial repetitive intergenic consensus-polymerase chain reaction; MDR, multidrug-resistant.

Our data also revealed that among the 370 *V. cholerae* isolates, ∼57.6% (*n* = 213) exhibited MDR phenotypes with 57 resistance profiles. Given that high incidence of heavy metal tolerant isolates was also observed, therefore, we further investigated the relationship between the MDR and heavy metal tolerance of the isolates. The 213 MDR isolates were classified into 11 distinct clusters (I to XI) with 148 ERIC-genotypes (Fig. 5). Approximately half of the MDR isolates were grouped into Cluster II (51.6%, *n* = 110) with 56 ERIC-genotypes. Cluster IX was the second largest cluster (16.4%, *n* = 35); it consisted of 35 MDR isolates with 29 ERIC-genotypes, followed by the Cluster III, Cluster VI, and Cluster VII containing 7.5% (*n* = 16), 7.0% (*n* = 15), and 6.1% (*n* = 13) of the MDR isolates, respectively. The remaining isolates (11.3%, *n* = 24) were classified into Clusters I, IV, V, VIII, X, and XI with percentages in the range from 3.3% to 0.5%.

Among the 213 MDR isolates, ∼40.4% (*n* = 86) were tolerant to 1 heavy metal, followed by 32.9% (*n* = 70), 14.1% (*n* = 30), and 3.8% (*n* = 8) to 2, 3, and 4 heavy metals, respectively. Various resistance profiles were observed in different phylogenetic clusters. For instance, the Hg/AMP/RIF/STR/SXT/TM resistance profile was predominant in the largest Cluster II (7.3%, 8/110), followed by Ni/Hg/AMP/RIF/SPT/STR/TET/TM (3.6%, 4/110), Hg/AMP/RIF/STR (2.7%, 3/110), Hg/AMP/RIF/SPT/STR/SXT/TET/TM (2.7%, 3/110), Ni/Hg/AMP/SPT/STR (2.7%, 3/110), and Cd/Hg/AMP/RIF/SPT/STR (2.7%, 3/110). In Cluster IX, about 22.9% (8/35) of isolates exhibited resistance to Cd/Hg/AMP/RIF/SPT/STR (11.4%, 4/35) and RIF/SPT/STR/SXT/TM (11.4%, 4/35). Some isolates in Cluster III had the Ni/STR/SXT/TM (18.8%, 3/16) and Ni/RIF/SXT/TM resistance profiles (12.5%, 2/16).

In addition, the MDR isolates with identical ERIC-genotypes had similar resistance profiles to antimicrobial agents, but different tolerance profiles to heavy metals. For instance, two isolates, *V. c-L. longirostris* 0805-18 and *V. c-P. pekinensis* 0807-08, recovered from *L. longirostris* and *P. pekinensis*, respectively, shared an identical ERIC-genotype Vc00008, and antibiotic resistance profile (AMP/RIF/STR), but former isolate resistant to Hg^2+^ and Ni^+^, and the latter only resistant to Hg^2+^.

Overall, these data demonstrated considerable genetic diversity of the 213 MDR *V. cholerae* isolates with various heavy metal tolerance phenotypes.

## Discussion

Outbreaks and prevalence of foodborne diseases not only are a major burden on global health care systems but also result in a huge negative impact on economic growth and social stability.^38^ *V. cholerae* is ubiquitous in aquatic environments worldwide. Continuous monitoring of *V. cholerae* contamination in aquatic products is crucial for assuming food safety. In this study, 370 *V. cholerae* strains were isolated and identified from the 12 species of aquatic products. Among these species, *I. punetaus*, *L. longirostris*, *O. argus Cantor*, and *P. fulvidraco* were for the first time analyzed for *V. cholerae*. The *I. punetaus* (known as channel catfish) is native to North America and its production reached 382,306 tons in 2017 in China.^29^ *L. longirostris* (known as longsnout catfish) is a rare and valuable freshwater economic fish in China, *O. argus Cantor* (known as snakehead fish) is also a popular freshwater fish and is regarded as a good nourishing tonic by Chinese people,^39^ and *P. fulvidraco* (known as yellow catfish) is a small fish that lives in shoals. The total output of these 3 species reached 227,454, 483,141, and 480,032 tons in 2017 in China.^29^

In this study, all the 370 *V. cholerae* isolates were absent for the toxic *ctxAB* and *tcpA* genes, consistent with our prior studies,^18,30,31^ and some other literature.^22,40–43^ The *ace*, *zot* genes within the CTX element of pathogenic *V. cholerae* were also absent from all the isolates tested in this study. A similar observation was also reported based on non-O1/O139 *V. cholerae* strains of environmental origins.^11,22,40,43^

Recently, Olivares *et al.* reported that non-O1/O139 *V. cholerae*, isolated from an 81-year-old woman with diarrheal disease, carried the *rtxA* gene.^44^ Within the *rtxCABD* operon, the *rtxA* gene encodes an RTX toxin, whereas the *rtxBD* and *rtxC* genes encode two secretion proteins and a toxin activator, respectively.^45^ In this study, the intact *rtxCABD* operon was observed in about 24.3% of the 370 *V. cholerae* isolates, which was remarkably lower than those of *rtxB^+^/rtxC^+^/rtxD^+^* genes (80.3%, 81.4%, and 80.8%, respectively). The lower occurrence of the *rtxA* gene (24.3%) was also reported by Schirmeister *et al.* (27.8%),^11^ but it was different from that (83.0%) by Xu *et al.*^18^ It will be interesting to investigate the possible mechanism whereby the *rtx* gene cluster is truncated in *V. cholerae* in future research.

The HlyA is a pore-forming toxin that causes ion leakage and, ultimately, eukaryotic cell lysis.^46^ Previous research has indicated that the HlyA and TLH prolonged *V. cholerae* colonization and pathogenesis to the epithelial cell.^47^ In this study, the *hlyA* and *tlh* genes were present in ∼81.4% and 80.5% of the *V. cholerae* isolates, respectively, consistent with the previous report.^18^ Ceccarelli *et al.* also reported that the *hlyA* gene was detected positive in 83% of *V. cholerae* isolates (*n* = 395) from oyster, sediment, and water samples collected from 2009 to 2012 in Maryland, United States.^14^ In addition, the *hapA* gene encoding a Zn-dependent hemagglutinin protease facilitates *V. cholerae* detachment from the intestinal mucosa.^48^ In this study, the *hapA* gene was present in ∼82.7% of the *V. cholerae* isolates, consistent with previous reports, such as 95.0% of the isolates (*n* = 400) from fish^18^ and 98% of the isolates (*n* = 794) from clinical and environmental samples.^49^ Meena *et al.* also reported that 46.5% of *V. cholerae* strains isolated from water samples in India carried the *hapA* gene.^50^

The MSHA is very important for surface colonization of *V. cholerae*.^51^ In this study, the *mshA* gene was detected positive in about 6.0% of the *V. cholerae* isolates, which was higher than that (0.8%) reported by Xu *et al.*,^18^ but lower than that (98.8%) by Rahman *et al.*^52^ The PilA, belonging to the putative Type IV pilus of *V. cholerae*, is a virulence factor for *Vibrio vulnificus*.^53^ In this study, the *pilA* gene was absent from all the 370 *V. cholerae* isolates tested, but this gene was present in 0.8% of the *V. cholerae* isolates in a previous report.^18^ Taken together, in this study, the *ctxAB*, *tcpA*, *ace*, *zot*, and *pilA* genes were absent from all the 370 *V. cholerae* isolates recovered from the 12 species of aquatic products; however, the other virulence-associated genes were prevalent (79.2–82.7%), such as *rtxBCD*, *hlyA*, *hapA*, and *tlh*.

It has been reported that China is the largest antibiotic producer and importer in the world.^54^ The inappropriate use of antimicrobial agents has resulted in high detection frequencies in surface water and sediment samples of lakes and rivers in China, for example, the Taihu Lake,^55^ the Yangtze River,^56^ and the Yellow River Delta.^57^ In this study, ∼60% of the *V. cholerae* isolates from the 12 species of aquatic products were resistant to STR and AMP. Xu *et al.* also reported that 44.5% and 65.3% of the *V. cholerae* isolates, recovered from four species of fish collected in 2017 in Shanghai, China, were resistant to AMP and STR, respectively.^18^ Moreover, in this study, ∼57.6% of the isolates exhibited MDR phenotypes with 57 resistance profiles, which were significantly different among the 12 species (MARI, *p* < 0.001). Notably, 20.3% of the *V. cholerae* isolates recovered from the fish samples showed MARI values >0.4, indicating that their sources posed a high risk of antimicrobial contamination.

Heavy metals are cytotoxic to humans at low concentrations.^58^ In this study, we observed that ∼69.5%, 32.4%, and 30.8% of the *V. cholerae* isolates recovered from the 12 species of aquatic products were tolerant to Hg^2+^, Ni^2+^, and Cd^2+^, respectively, when compared with the quality control strain. Tolerant *V. cholerae* isolates to the three heavy metals were more than those (Hg^2+^, 49.3%; Ni^2+^, 0.3%; and Cd^2+^, 4.8%) in the previous report.^18^ Wang *et al.* investigated the circumstances of a rice-fish-farming system, and they found that Pb, Cd, Hg, As, and Cr constituted the most severe toxic heavy metal pollution in south China.^59^ In this study, only two isolates (*V. c*- *S. maximus* 0706-87, *V. c*-*C. auratus* 0709-17) showed tolerance to Pb^2+^, but none to Cr^3+^. In addition, 41.6% of the 370 *V. cholerae* isolates were tolerant to 2 or more heavy metals, of which notably 9 *V. cholerae* isolates were tolerant to 4 heavy metals. Different heavy metal tolerance profiles were also observed among the *V. cholerae* isolates from the 12 species of aquatic products. These results suggested that various levels of heavy metal pollution may exist in aquatic farming environments.

The ERIC sequences are intergenic repetitive units in *E. coli* and other members of the Enterobacteriaceae, such as *V. cholerae.*^60^ The ERIC-PCR has been applied in clonal diversity and genotyping of *V. cholerae* isolates from clinical and environmental origins.^6,18,61,62^ For instance, Xu *et al.* reported 328 ERIC-genotypes among 400 *V. cholerae* isolates derived from 4 species of fish.^18^ In this study, the 370 *V. cholerae* isolates recovered from the 12 species of aquatic products were classified into 239 ERIC-genotypes, 69.9% of which were assigned as singletons. These data, coupled with previous studies, demonstrated considerable genetic diversity of *V. cholerae* in aquatic products.

In conclusion, in this study, 370 *V. cholerae* isolates were isolated from the 12 species of commonly consumed aquatic products, which were collected from the two large aquatic product markets in Shanghai, China, from July to September of 2018. Among these species, *V. cholerae* was for the first time detected in the species *I. punetaus*, *L. longirostris*, *O. argus Cantor*, and *P. fulvidraco*. The CT genes *ctxAB* and *tcpA*, as well as toxin-associated genes *ace*, *zot*, and *pilA* were absent from all the 370 *V. cholerae* isolates, indicating no contamination of epidemic *V. cholerae* strains in the aquatic product samples tested. Nevertheless, high detection frequencies of the other virulence-associated genes were observed among the isolates, including the *hapA* (82.7%), *hlyA* (81.4%), *rtxCABD* (81.4%, 24.3%, 80.3%, and 80.8%, respectively), and *tlh* (80.5%).

Meanwhile, ∼62.2% of the 370 *V. cholerae* isolates were resistant to STR, followed by AMP (60.3%), RIF (53.8%), TM (38.4%), and SXT (37.0%). Approximately 57.6% of the isolates exhibited MDR phenotypes with 57 resistance profiles, which was significantly different among the 12 species (MARI, *p* < 0.001). High incidence of tolerance to heavy metals Hg^2+^ (69.5%), Ni^2+^ (32.4%), and Cd^2+^ (30.8%) was observed among the isolates. Approximately 17.4% of the MDR isolates were tolerant to three or four heavy metals, implying a high risk of antibiotic and heavy metal contamination in the aquaculture environment for the aquatic animals tested. The obtained ERIC-PCR fingerprinting profiles classified all the 370 *V. cholerae* isolates into 239 different ERIC-genotypes, and 69.9% thereof were assigned as singletons, which demonstrated considerable genomic variation among the isolates.

In future research, it will be interesting to further investigate coevolution mechanisms of the antibiotic resistance and heavy metal tolerance of *V. cholerae* isolates in aquatic products. Overall, this study provided data in support of food safety risk assessment of aquatic products, and aquatic animal health management in aquaculture industry.

## Supplementary Material

Supplemental data

Supplemental data

## Notes

^†^ https://www.who.int/
