# Supplementary material for: Virulence, Resistance, and Genomic Fingerprint Traits of Vibrio cholerae Isolated from 12 Species of Aquatic Products in Shanghai, China
Source: Microb Drug Resist. 2020 Dec 3;26(12):1526–39. doi: 10.1089/mdr.2020.0269 (PMC7757592; doi:10.1089/mdr.2020.0269)
Supplement: Supplemental data [file Suppl_TableS1.docx]

Table S1 Antibiotic resistant *V. cholerae* isolates in the twelve species of aquatic products

| Sample | Species | No. of isolates | Resistant isolates (%) | | | | | | | | | | Highest  MARI |
| --- | --- | --- | --- | --- | --- | --- | --- | --- | --- | --- | --- | --- | --- |
|  |  |  | AMP | CHL | CN | KAN | RIF | SPT | STR | SXT | TET | TM |  |
| Fish | *A. nobilis* | 30 | 43.3 | 0.0 | 0.0 | 0.0 | 50.0 | 23.3 | 56.7 | 23.3 | 10.0 | 23.3 | 0.6 |
|  | *C. auratus* | 30 | 53.3 | 3.3 | 6.7 | 16.7 | 60.0 | 63.3 | 90.0 | 73.3 | 10.0 | 23.3 | 0.7 |
|  | *C. idellus* | 30 | 100.0 | 0.0 | 10.0 | 6.7 | 100.0 | 46.7 | 80.0 | 50.0 | 76.7 | 56.7 | 0.8 |
|  | *I. punetaus* | 80 | 88.8 | 0.0 | 1.3 | 2.5 | 78.8 | 31.3 | 58.8 | 27.5 | 3.8 | 26.3 | 0.6 |
|  | *L. longirostris* | 10 | 70.0 | 0.0 | 0.0 | 0.0 | 50.0 | 10.0 | 100.0 | 30.0 | 0.0 | 30.0 | 0.4 |
|  | *O. argus cantor* | 40 | 57.5 | 0.0 | 0.0 | 0.0 | 12.5 | 12.5 | 42.5 | 35.0 | 0.0 | 45.0 | 0.5 |
|  | *P. fulvidraco* | 14 | 7.1 | 0.0 | 0.0 | 7.1 | 35.7 | 21.4 | 71.4 | 85.7 | 0.0 | 92.9 | 0.5 |
|  | *P. pekinensis* | 30 | 70.0 | 0.0 | 3.3 | 6.7 | 36.7 | 33.3 | 90.0 | 0.0 | 0.0 | 0.0 | 0.5 |
|  | *S. maximus* | 64 | 42.2 | 0.0 | 0.0 | 1.6 | 40.6 | 35.9 | 60.9 | 34.4 | 1.6 | 34.4 | 0.5 |
| Shellfish | *O. gigas thunberg* | 14 | 85.7 | 0.0 | 0.0 | 21.4 | 71.4 | 35.7 | 92.9 | 78.6 | 0.0 | 78.6 | 0.7 |
|  | *P. magellanicus* | 16 | 0.0 | 0.0 | 0.0 | 6.3 | 50.0 | 31.3 | 93.3 | 18.8 | 0.0 | 25.0 | 0.6 |
| Shrimp | *P. vannamei* | 12 | 8.33 | 0.0 | 0.0 | 0.0 | 33.3 | 16.7 | 25.0 | 8.3 | 8.3 | 8.3 | 0.7 |
