# Supplementary material for: Virulence, Resistance, and Genomic Fingerprint Traits of Vibrio cholerae Isolated from 12 Species of Aquatic Products in Shanghai, China
Source: Microb Drug Resist. 2020 Dec 3;26(12):1526–39. doi: 10.1089/mdr.2020.0269 (PMC7757592; doi:10.1089/mdr.2020.0269)
Supplement: Supplemental data [file Suppl_TableS2.docx]

Table S2 Heavy metal tolerant *V. cholerae* isolates in the twelve species of aquatic products

| Sample | Species | No. of isolates | Resistant isolates (%) | | | | | | | |
| --- | --- | --- | --- | --- | --- | --- | --- | --- | --- | --- |
|  |  |  | Cd^2+^ | Cr^3+^ | Cu^2+^ | Hg^2+^ | Mn^2+^ | Ni^2+^ | Pb^2+^ | Zn^2+^ |
| Fish | *A. nobilis* | 30 | 30.0 | 0.0 | 0.0 | 63.3 | 0.0 | 23.3 | 0.0 | 0.0 |
|  | *C. auratus* | 30 | 36.3 | 0.0 | 3.3 | 63.3 | 0.0 | 10.0 | 3.3 | 0.0 |
|  | *C. idellus* | 30 | 0.0 | 0.0 | 0.0 | 80.0 | 3.3 | 90.0 | 0.0 | 30.0 |
|  | *I. punetaus* | 80 | 45.0 | 0.0 | 1.3 | 92.5 | 0.0 | 36.3 | 0.0 | 11.3 |
|  | *L.longirostris* | 10 | 40.0 | 0.0 | 0.0 | 90.0 | 0.0 | 90.0 | 0.0 | 10.0 |
|  | *O. argus Cantor* | 40 | 45.0 | 0.0 | 5.0 | 52.5 | 0.0 | 22.5 | 0.0 | 7.5 |
|  | *P. fulvidraco* | 14 | 0.0 | 0.0 | 0.0 | 14.3 | 0.0 | 9.3 | 0.0 | 0.0 |
|  | *P. pekinensis* | 30 | 20.0 | 0.0 | 0.0 | 83.3 | 0.0 | 30.0 | 0.0 | 0.0 |
|  | *S. maximus* | 64 | 17.2 | 0.0 | 0.0 | 46.9 | 0.0 | 15.6 | 1.6 | 4.7 |
| Shellfish | *O. gigas Thunberg* | 14 | 35.7 | 0.0 | 0.0 | 92.9 | 0.0 | 0.0 | 0.0 | 7.1 |
|  | *P.magellanicus* | 16 | 18.8 | 0.0 | 0.0 | 68.8 | 0.0 | 18.8 | 0.0 | 0.0 |
| Shrimp | *P. vannamei* | 12 | 75.0 | 0.0 | 0.0 | 83.3 | 0.0 | 0.0 | 0.0 | 0.0 |
